# Supplementary material for: Causal Relationship between Aging and Anorexia Nervosa: A White-Matter-Microstructure-Mediated Mendelian Randomization Analysis
Source: Biomedicines. 2024 Aug 16;12(8):1874. doi: 10.3390/biomedicines12081874 (PMC11351342; doi:10.3390/biomedicines12081874)
Supplement: Supplementary file 1 [file biomedicines-12-01874-s001.zip › Supplementary Figure/Figure S4/Singe SNP plot of MR analysis for TL to ubm-b-1726.pdf]

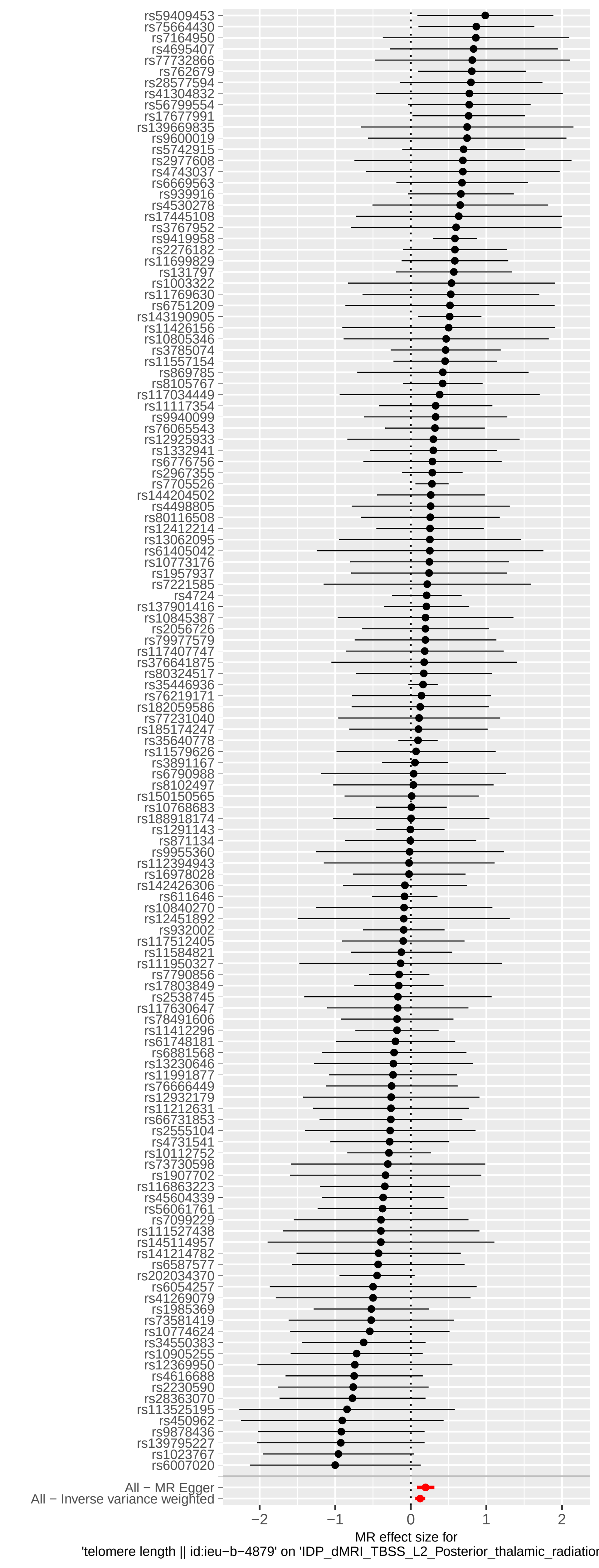

rs59409453
rs75664430
rs7164950
rs4695407
rs77732866
rs762679
rs28577594
rs41304832
rs56799554
rs17677991
rs139669835
rs9600019
rs5742915
rs2977608
rs4743037
rs6669563
rs939916
rs4530278
rs17445108
rs3767952
rs9419958
rs2276182
rs11699829
rs131797
rs1003322
rs11769630
rs6751209
rs143190905
rs11426156
rs10805346
rs3785074
rs11557154
rs869785
rs8105767
rs117034449
rs11117354
rs9940099
rs76065543
rs12925933
rs1332941
rs6776756
rs2967355
rs7705526
rs144204502
rs4498805
rs80116508
rs12412214
rs13062095
rs61405042
rs10773176
rs1957937
rs7221585
rs4724
rs137901416
rs10845387
rs2056726
rs79977579
rs117407747
rs376641875
rs80324517
rs35446936
rs76219171
rs182059586
rs77231040
rs185174247
rs35640778
rs11579626
rs3891167
rs6790988
rs8102497
rs150150565
rs10768683
rs188918174
rs1291143
rs871134
rs9955360
rs112394943
rs16978028
rs142426306
rs611646
rs10840270
rs12451892
rs932002
rs117512405
rs11584821
rs111950327
rs7790856
rs17803849
rs2538745
rs117630647
rs78491606
rs11412296
rs61748181
rs6881568
rs13230646
rs11991877
rs76666449
rs12932179
rs11212631
rs66731853
rs2555104
rs4731541
rs10112752
rs73730598
rs1907702
rs116863223
rs45604339
rs56061761
rs7099229
rs111527438
rs145114957
rs141214782
rs6587577
rs202034370
rs6054257
rs41269079
rs1985369
rs73581419
rs10774624
rs34550383
rs10905255
rs12369950
rs4616688
rs2230590
rs28363070
rs113525195
rs450962
rs9878436
rs139795227
rs1023767
rs6007020
All − MR Egger
All − Inverse variance weighted
−2
−1
0
1
2
MR effect size for
'telomere length || id:ieu−b−4879' on 'IDP_dMRI_TBSS_L2_Posterior_thalamic_radiatio
